# Quality Parameters of Juice Obtained from Hydroponically Grown Tomato Processed with High Hydrostatic Pressure or Heat Pasteurization

**DOI:** 10.1155/2020/4350461

**Published:** 2020-09-04

**Authors:** Maja Jeż, Wioletta Błaszczak, Kamila Penkacik, Ryszard Amarowicz

**Affiliations:** Institute of Animal Reproduction and Food Research, Polish Academy of Sciences, Tuwima 10, 10-748 Olsztyn, Poland

## Abstract

The effect of processing such as high hydrostatic pressure (HHP) (400-600 MPa/15 min) or low pasteurization temperature (LPT) (74°C/2 min) or high pasteurization temperature (HPT) (90°C/1 min) on selected quality parameters of juice obtained from hydroponically cultivated beef tomatoes was investigated. The total polyphenols content (TPC), total phenolic index (TPI), Trolox equivalent antioxidant capacity (ABTS) and ferric reducing antioxidant power (FRAP) were analysed in the fresh and processed juices stored for 0, 7 and 14 days. What is more, colour parameters (*L*^∗^, *a*^∗^, *b*^∗^, *∆E*), the activity of polyphenol oxidase (PPO) and peroxidase (POD) and microbial stability were also analyzed following the juices storage. Among all the tested samples, the juice exposed to 600 MPa for 15 min showed superior quality. Samples treated with 600 MPa for 15 min and stored for 0, 7 and 14 days had high TPC, TPI, ABTS, FRAP and *a*^∗^ values. As demonstrated, these tested samples at the end of the storage period retained 90% and 95% of their polyphenol content and antioxidant capacity, respectively. As in the case of pasteurization, juice processing at 600 MPa for 15 min clearly reduced the activity of food-spoiling enzymes (PPO, POD) as well as the microbial count. The obtained results showed that TPC was significantly and positively correlated with TPI, ABTS and FRAP parameters.

## 1. Introduction

Tomato polyphenols are known to contribute significantly to the nutritional quality of tomato-based products [1]. The activity of plant enzymes such as polyphenol oxidase (PPO) and peroxidase (POD) results in the degradation of polyphenols, leading to browning discolouration particularly in fresh or lightly processed products [2]. Almost 60% of the annual production of tomatoes is highly processed into ketchups, juices, puree, or other preserves. Processing involving temperature treatment distinctly enhances the enzymatic and microbiological stability of tomato-based products. On the other hand, it may lead to substantial changes in the quantity and quality of thermolabile phytochemicals [3]. Tomato pulp subjected to pasteurization demonstrated 15% and 20% decreases in rutin and chlorogenic acid contents, respectively. However, the content of naringenin chalcone was completely lost after the canning process and the pasteurization (93°C) of the tomato pulp [4]. L-ascorbic acid is likely one of the more reactive compounds present in tomato; thus, it is particularly vulnerable to thermal processing [5]. Thermal treatment not only results in the loss of labile bioactive compounds but also may affect product properties such as flavour, colour, or texture. As indicated, thermally treated tomato juices showed undesirable changes in colour parameters [6, 7]. The distinct changes in *L*^∗^, *a*^∗^, *b*^∗^ or Δ*E* values observed in the cited above reports for heat-treated samples resulted not only from the formation of Maillard reaction products but also from decomposition of thermolabile phytochemicals, which occurred upon processing.

Compared to heat treatment, HHP provides better retention of antioxidants from the food matrix already at ambient temperature. What is more, the polyphenols of fruits or vegetables as well as their antioxidant properties can be preserved more effectively by high hydrostatic pressure (HHP) than by thermal treatment [8]. Considering above, HHP processing can be an alternative for heat treatment in the context of food quality and nutritional value. Tomato juice (pH 3.9) exposed to 600 MPa for 1 min demonstrated a higher total phenolic content (20%) and antioxidant activity (24%) than thermally treated tomato juice [9]. A tomato sample treated at 600 MPa for 15 min showed higher (by almost 9%) total phenolic content and higher (by 38%) antiradical power as compared to the thermally processed puree [10]. What is more, a number of attempts have been made to use HHP instead of high-temperature treatments to inactivate food-spoiling microorganisms [11, 12] and unwanted food enzymes [13]. The results obtained by Terefe et al. [2] indicated that the optimal conditions to inactivate POD in strawberries were 600 MPa at 60°C for 10 min, but no substantial PPO inactivation was noted when these conditions were applied. Marszałek et al. [14] showed that pressurization (500 MPa) of strawberry puree at 50°C after 15 min was required to achieve 72% and 50% PPO and POD inactivation, respectively.

As indicated, some critical aspects regarding plant variety selection and the conditions of the HHP process must be considered when applying HHP to obtain novel products with high nutritional quality [15].

The effects of HHP processing on the quality of tomato juice have been extensively studied in the literature [1, 9, 15]. However, the quality parameters of tomato-based products obtained from fruit grown in a hydroponic system remain insufficiently investigated. Currently, hydroponic systems are considered to be one of the most profitable and popular systems in crop production [16]. On the other hand, hydroponic crops may significantly differ in terms of their quality parameters, especially in terms of the content of antioxidants or the activity of food spoiling enzymes, compared to crops obtained using conventional systems. Therefore, this study evaluated the effect of HHP treatment (400-600 MPa/15 min) on the phenolic content, antioxidant capacity, colour parameters, and PPO and POD activity as well as the microbial stability of juice obtained from hydroponically cultivated beef tomatoes. All the aforementioned evaluations were also performed on juices subjected to a low pasteurization temperature (LPT, 74°C/2 min) or a high pasteurization temperature (HPT, 90°C/1 min). The quality parameters of the tomato juices treated with HHP, LPT, and HPT were also examined upon sample storage at 6 ± 2°C for 0, 7, and 14 days.

## 2. Material and Methods

### 2.1. Chemicals

All solvents used were HPLC- or analytical-grade unless otherwise specified. 2,2′-Azinobis (3-ethylbenzothiazoline-6-sulfonic acid) diammonium salt (ABTS); 6-hydroxy-2,5,7,8-tetramethylchroman-2-carboxylic acid (Trolox); 2,4,6-tris(pyridyl-s-triazine) (TPTZ); Folin and Ciocalteu's phenol reagent; gallic, ferulic, and *p*-coumaric acids; polyvinylpyrrolidone (PVPP, ~110 *μ*m); catechol (>99%); hydrogen peroxide (30%); and Triton X-100 were obtained from Sigma Chemical Co. (Poznań, Poland). The remaining reagents (all of reagent-grade quality) were supplied by POCh (Gliwice, Poland). Water was purified using the Milli-Q system (Millipore, Bedford, USA).

### 2.2. Sample Preparation

The mature tomatoes of the *Beef* variety grown in a hydroponic system were purchased from the Łegajny greenhouse complex, Łegajny, Poland. The juice was produced from fresh fruits using a juicer (domestic appliance, BOSCH CNCJ04, Germany) equipped with a ceramic blade. The seed and skin were automatically separated upon juice processing. The fresh juice was directly subjected to high hydrostatic pressure treatment or thermal pasteurization.

### 2.3. High Hydrostatic Pressure (HHP) Process

The juice samples were enclosed in Teflon tubes (50 mL), deaerated, tightly sealed, and subjected to HHP using a high-pressure system (Unipress U-303, Warsaw, Poland). The Teflon tubes were put into a high-pressure chamber with a capacity of approximately 100 mL filled with a pressure-transmitting medium (water-propylene glycol (propane-1,2-diol), 1 : 1, *v*/*v*), which also minimized adiabatic heating. The samples were pressure-treated at 400 or 600 MPa for 15 min. The compression and decompression rates were 8 MPa/s and 10 MPa/s, respectively. The sample temperature reached 32 ± 1.5°C at a pressure of 400 MPa and 38 ± 1.5°C at a pressure of 600 MPa. Data acquisition software (OMEGASOFT, OMB DAQ-54, OMEGA Inc., Hungary) was used to collect all the parameters of the HHP treatment. The analyses were carried out in triplicate for each treatment.

### 2.4. Pasteurization Process

The juice samples (250 mL) were placed in glass bottles, and the low pasteurization temperature (LPT) (74 ± 2°C/2 min) or the high pasteurization temperature (HPT) (90 ± 1°C/1 min) was applied in a water bath (IKA, HBR4 Digital, Germany).

A thermocouple positioned at the juice cold point was used to control the temperature. The time for the juice to reach the required temperature was less than 5 min. Once the pasteurization temperature was reached, the juice samples were immediately cooled to room temperature by immersing the bottle in a water/ice mixture. The analyses were carried out in triplicate for each treatment.

### 2.5. Storage Condition

Nonprocessed tomato juice was used as a reference sample. The untreated and HHP-, LPT-, and HPT-treated juices were stored at 6 ± 2°C in the dark and analysed after 0, 7, and 14 days of storage.

### 2.6. Extraction Procedure

The juices were lyophilized prior to the extraction of phenolic compounds (Labcono 195, England), and the extraction procedure was performed using a water : methanol mixture (20 : 80, *v*/*v*). The juice powder and the extraction mixture (1 : 20, *w*/*w*) were treated at 70°C for 15 min under continuous shaking using a water bath (Julabo SW 22, Germany). The process was repeated three times. The extracts obtained were combined, filtered through filter paper, and concentrated under reduced pressure on a rotary evaporator (Buchi Rotavapor R-200, Switzerland) with a warm water bath at 54 ± 2°C and lyophilized. The freeze-dried extracts were stored at -24°C until analysis.

### 2.7. Total Phenolic Content (TPC)

The number of phenolic compounds in the juice extract was measured using Folin and Ciocalteu's phenol reagent [17]. After colour development, the absorbance was measured at 725 nm (Beckman DU® 7500 spectrophotometer, California, USA) after sample incubation (TH-24 block heater, Meditherm, Poland) at 20°C for 20 min. The results were expressed as mg of gallic acid (GAE)/g of juice dry matter (dm). The linearity range of the calibration curve was from 0.062 to 0.50 mg/mL (*R*^2^ = 0.999).

### 2.8. Reversed-Phase High-Performance Liquid Chromatography (RP-HPLC)

For the RP-HPLC fingerprint analysis of individual phenolic compounds present in the tomato juice extract, a Shimadzu system (Shimadzu Corp., Kyoto, Japan) consisting of two LC-10AD pumps, an SCTL 10A system controller, an SPD-M 10 A photodiode array detector, and a prepacked LUNA C 18 column (4 × 259 mm, 5 *μ*m, Phenomenex) was used. A flow rate of 1 mL/min, injection volume of 20 *μ*L, a gradient elution of acetonitrile-water-acetic acid (5 : 93 : 2, *v*/*v*/*v*) (solvent A) and acetonitrile-water-acetic acid (40 : 58 : 2, *v*/*v*/*v*) (solvent B), and a 0-50 min solvent B from 0% to 100% were applied [18]. The tomato juice extract was dissolved using a water : methanol mixture (20 : 80, *v*/*v*) and filtered through a 0.45 *μ*m filter (Chromafil Xtra PET-45/25, Macherey-Nagel). The separation of compounds was monitored at 260 and 320 nm. The identification was performed based on the retention times and the UV spectra of the standards and the samples.

### 2.9. Trolox Equivalent Antioxidant Capacity (ABTS^·+^)

The method described by Re et al. [19] was used to determine the TEAC of tomato juice extracts.

To perform the measurements, the ABTS^·+^ solution was diluted with a water : methanol mixture (20 : 80, *v*/*v*) to an absorbance level of 0.70 ± 0.02 at 734 nm. For the spectrophotometric assay, 1.48 mL of the ABTS^·+^ solution and 20 *μ*L of the respective extract, Trolox or blank (80% methanol), were mixed, and absorbance was measured at 734 nm (Beckman DU® 7500 spectrophotometer, California, USA) directly after sample incubation (TH-24 block heater, Meditherm, Poland) at 30°C for 6 min. The results were expressed as *μ*mol Trolox equivalents/g of juice dm. The linearity range of the calibration curve was from 0.0 to 2.0 mM (*R*^2^ = 0.999).

### 2.10. Ferric Reducing Antioxidant Power (FRAP)

The FRAP assay was carried out according to the procedure described by Benzie and Strain [20]. A Fe^3+^-TPTZ complex was generated at pH 3.6 (300 mM acetate buffer) by mixing 10 mM TPTZ in 40 mM HCl and 20 mM ferric chloride (1 : 1, *v*/*v*). Then, 75 *μ*L of tomato juice extract or blank (80% methanol) and water (225 *μ*L) were added to the complex solution (2.25 mL). The absorbance was read at 593 nm (Beckman DU® 7500 spectrophotometer, California, USA) after sample incubation (TH-24 block heater, Meditherm, Poland) at 37°C for 30 min. Trolox was used to prepare a calibration curve (from 0.0 to 1.0 mM; *R*^2^ = 0.999). The results were expressed as *μ*mol Trolox equivalents/g of juice dm.

### 2.11. Colour Measurements

The colour of fresh, untreated, HHP-, LPT-, and HPT-processed tomato juices at 0, 7, and 14 days of storage was analysed using the colour space ColorFlex (HunterLab, USA). The following colour parameters were determined: *L*^∗^ (represents lightness, where *L*^∗^ = 0 (black) and *L*^∗^ = 100 (white)), *a*^∗^ (‐*a*^∗^ = greenness and +*a*^∗^ = redness), and *b*^∗^ (‐*b*^∗^ = blueness and +*b*^∗^ = yellowness). A standard white and black plate was used to calibrate the instrument before analysis. The obtained results were expressed in accordance with the CIELab system with reference to the standard illuminant D65 and a visual angle of 10° [21].

Additionally, in the processed and stored tomato juice, the total colour differences (Δ*E*) were calculated using the following equation [22]:

(1) $$\begin{array}{c} \Delta E={\left({\left(\Delta L\right)}^{2}+{\left(\Delta a\right)}^{2}+{\left(\Delta b\right)}^{2}\right)}^{1/2}, \end{array}$$

where Δ*L*, Δ*a*, and Δ*b* are the differences in *L*^∗^, *a*^∗^, and *b*^∗^ values between the untreated (control) sample and the treated sample on a particular day of storage.

### 2.12. Determination of PPO and POD Activities

The PPO and POD activities were determined as described by Marszałek et al. [14]. The selected tissue enzymes were extracted using 0.2 mol/L phosphate buffer (pH = 6.5) containing polyvinylpyrrolidone (40 mg/cm^3^), Triton X-100 (10 mg/cm^3^), and 1 mol/L NaCl. The juice and the extraction mixture (1 : 1, *w*/*w*) were treated with ultrasound (50 Hz, 25°C, ULTRON, Poland) for 3 min and centrifuged (MPW 350R, MPW Med. Instrument, Poland) at 17,700 g for 30 min at 4°C, and the obtained supernatant was filtered through blotting paper.

For the PPO activity assay, 100 *μ*L of the supernatant was mixed with 3 mL of 0.05 mol/L phosphate buffer (pH = 6.5) containing 0.07 mol/L catechol.

For the POD activity assay, 1.5 mL of 0.05 mol/L phosphate buffer (pH = 6.5) was added to 200 *μ*L of the supernatant, 200 *μ*L of 0.05 mol/L phosphate buffer containing p-phenylenediamine (10 mg/cm^3^), and 200 *μ*L of hydrogen peroxide (16.6 mg/cm^3^).

The absorbance of the mixture was measured at 420 nm (for the PPO assay) and at 485 nm (for the POD assay) at 25°C for 15 min using a UV-visible spectrophotometer (UNICAM He*λ*ios Alpha & Beta, Cambridge, UK).

A blank sample was prepared in the same way by substituting the supernatant with 0.2 M phosphate buffer. The PPO and POD activity was expressed as a change in absorbance at the respective wavelength *Δ*OD/min/g of fresh weight (fw) of the analysed sample.

The residual activity (%) of the enzymes studied was calculated as follows:

(2) $$\begin{array}{c} \text{The residual activity}\left(\%\right)=\left(\frac{A}{A_{0}}\right)\times 100\%, \end{array}$$

where *A*_0_ represents the activity of the enzyme in the untreated (reference) sample and *A* represents the enzyme activity in the treated sample.

### 2.13. Microbiological Evaluation

The total aerobic mesophilic count (TAMC), total yeast count (TYC), and total mould count (TMC) were analysed in the fresh, untreated juice and in the juices subjected to processing (HHP, LPT, and HTP) upon storage at 6 ± 2°C for periods of 0, 7, and 14 days.

A sample of juice (10 mL) was diluted with 90 mL of saline peptone (SP, 1 g/L peptone, 8.5 g/L NaCl) and homogenized in a stomacher (Model 400, Seward, London, UK) at a regular speed for 2 min.

To determine the TAMC of the juice samples, the homogenates were serially diluted and plated on plate count agar (Merck, No. 105463), followed by incubation at 30 ± 1°C for 3 days. Mould and yeasts were determined by plating the homogenates in dichloran rose bengal chloramphenicol agar (Merck, No. 100466), followed by incubation at 25 ± 1°C for 5 days.

After incubation, the plates were counted, and the results were expressed as colony-forming units per 1 mL (CFU/mL). The determinations were carried out in triplicate. The detection limit was <1 CFU/mL.

### 2.14. Statistical Analysis

Data are presented as the mean ± standard deviation (SD) and were processed statistically using Statistica 13 software (Statsoft, USA). Differences between the obtained data were investigated by one-way analysis of variance (ANOVA) and Tukey's test (*p* ≤ 0.05). Pearson's correlation analysis was carried out to determine the correlation between all the studied parameters (TPC, TPI, ABTS, FRAP, *L*^∗^, *a*^∗^, *b*^∗^, Δ*E*, PPO, and POD). Principal component analysis (PCA) was also carried out to analyse the original variables (TPC, TPI, ABTS, FRAP, *L*^∗^, *a*^∗^, *b*^∗^, Δ*E*, PPO, and POD) and to replace them with the appropriate number of components. The PCA was based on a correlation matrix.

## 3. Results and Discussion

### 3.1. The Effects of Processing on the Total Phenolic Content (TPC) and the Total Phenolic Index (TPI)

The total phenolic content (TPC) obtained for methanolic extracts of tomato juices subjected to HHP (400-600 MPa/15 min), LPT (74°C/2 min), and HPT (90°C/1 min) processing is presented in Table 1.

The TPC measured in the extract obtained from the untreated tomato juice constituted 7.08 mg GAE/g dm. The content of phenolics presented in our study is comparable to the data obtained for juices produced from conventionally grown tomatoes [23]. As noted by these authors, the TPC found in commercially available tomato juices ranged from approximately 2.0 to 9.4 mg GAE/g dm. On the other hand, the concentration of polyphenols in the fresh, untreated juice was almost 30% and 40% higher than that found in hydroponically grown tomatoes [24, 25]. However, as demonstrated in the literature, the plant genotype and growth conditions are key determinants of the nutritional quality of the obtained product [26].

As shown in our study, pressurization did not significantly modify polyphenol content in the tested juices. In contrast, the fresh juices subjected to LPT and HPT yielded 21% and 31% reductions in TPC, respectively. Despite such a marked decrease, the polyphenol concentration in the tested juices did not differ significantly from that (4.59-8.67 mg gallic acid/g juice, dm) measured in commercially available, heat-pasteurized juices [27]. Our results are also in accordance with another reference in which a 22% decrease in TPC was observed after juice processing at 90°C for 1 min [9]. Similarly, only a slight variation (6% decrease) in TPC was found by these authors after juice treatment with HHP (600 MPa/1 min).

Among all the treated and stored (for 7 and 14 days) samples, the juice exposed to 600 MPa/15 min showed the highest TPC (Table 1). The value obtained for the tested juice on day 14 of storage was more than 10% and 30% higher than the values noted for the LPT- and HPT-treated juices, respectively. The obtained results clearly indicated that the HPT-treated juice showed a distinct reduction in TPC (by 15%) compared to the untreated sample. In contrast to our results, Vallverdu-Queralt et al. [23] found a minor variation (2-5% drop) in TPC in pasteurized tomato juices stored for 3 months.

Considering the results obtained for individual samples (Table 1), it is evident that LPT- and HPT-treated juices showed slight, insignificant variations in TPC throughout the entire storage period. At the end of the storage period, the juices exposed to HHP processing had 21% and 10% lower TPC values than those of the fresh samples exposed to 400 MPa/15 min and 600 MPa/15 min, respectively.

A more substantial drop (by 29% and 39%) in TPC was noted in the pasteurized and HHP-treated juices, respectively, after 1 month of storage [9]. Considering the above, it is evident that not only the processing and storage conditions but also the raw material used in the production step substantially affected the obtained values.

The total phenolic index (TPI) was quantified as the sum of the individual components identified in the analysed extracts using HPLC by comparing their retention time and UV spectra against the reference standards (Table 1).

As a result of processing, TPI decreased by 9% and 18% in the LPT- and HPT-treated juices, respectively, compared to that in the untreated sample (153.57 *μ*g/100 g dm). However, the pressurized juices showed no substantial changes in TPI values. Similarly, after 7 days of storage, the juices exposed to HHP or LPT treatment showed no significant changes in TPI values compared to those in the untreated sample. In contrast, an almost 40% drop in TPI was noted in the HPT-treated juice during that storage period. Similarly, almost half of the polyphenol concentration in the HPT-treated juice was lost after 14 days of storage. However, the pressurized juices had 9% and 48% higher TPI values (on average) than those noted for the reference and HHP-treated juices, respectively. A similar trend was observed in the work of Dede et al. [6]. In the cited study, the heat-treated juice demonstrated a 70% loss of antioxidants during 30 days of storage, whereas only slight changes were noted in the stored and HHP-treated juices. The polyphenol quantification by HPLC showed clearly that only the HHP-treated juices maintained individual compounds at high levels during the entire storage period.

### 3.2. The Effects of Processing on Antioxidant Capacity

The antioxidant capacities of the hydrophilic extracts of the analysed tomato juices determined in the ABTS and FRAP tests are presented in Table 1.

The ABTS value obtained for the juices exposed to HHP or LPT did not change significantly compared to that of the untreated juice (8.44 *μ*mol Trolox/g juice dm) (Table 1). In contrast, the antioxidant capacity of the HPT-treated juice was approximately 26% lower than the value noted for the reference sample. Similarly, Jayathunge et al. [9] found that pressurized (600 MPa/1 min) fresh tomato juices successfully retained their capacity to scavenge ABTS radicals. These authors also found that thermally processed (90°C/2 min) juice showed a significant reduction (by approximately 15%) in ABTS values.

In the first 7 days of storage, the pressurized (400 MPa/15 min) juice and LPT-treated juice showed nonsignificant variation in their ABTS values compared to those of the stored reference material (Table 1). However, a 15% drop in antioxidant capacity was noted for the HPT-treated stored juices. Among all the samples tested after 7 days of storage, the juice processed at 600 MPa/15 min had the highest ABTS value. After 14 days of storage, the juices treated with HHP and LPT were characterized by similar, still relatively high ABTS values, which were on average 40% higher than the value noted in the HPT juice.

We also found no changes in the ABTS value of the LPT-treated juice after 14 days of storage compared to the fresh, pasteurized juice. Similarly, 92% of the antioxidant potential of the juice exposed to 600 MPa/15 min was retained. However, the juices treated with 400 MPa/15 min and HPT retained 85% (on average) of their ability to scavenge ABTS radicals. As found by Fernandez Garcia et al. [28], tomato puree exposed to HHP treatment (500-800 MPa/5 min) retained 60-70% of its antioxidant capacity after 21 days of storage.

The antioxidant capacity of the analysed juice extracts determined in the FRAP assay (Table 1) showed clearly that the juice processed with 600 MPa for 15 min had the highest FRAP values of all the analysed samples. The tested juices retained 95% of their reducing power at the end of the storage period. Similarly, the HPT-treated juice also showed stable FRAP values during storage. However, its scavenging power was 6% lower than that found for the HHP-treated (600 MPa/15 min) juices. It was also found that pressurized (250 MPa/15 min) juice stored for 30 days had approximately 40% higher total antiradical capacity than the heat-pasteurized (80°C/1 min) sample [6]. Among all the processed juices, the greatest decrease (by 13%) in FRAP values was noted for the LPT juice after 14 days of storage.

### 3.3. The Effects of Processing on the Colour Parameters

In this study, colour parameters such as *L*^∗^ (lightness), *a*^∗^ (redness), and *b*^∗^ (yellowness) were recorded for the fresh, untreated juice and for the juices subjected to HHP, LPT, and HPT processing upon storage for 0, 7, and 14 days. The obtained parameters were also used to calculate the Δ*E* coefficient to estimate the total colour change as a result of processing and storage (Table 2). As shown in the literature, the Δ*E* coefficient clearly characterizes total colour changes in the processed products, from not noticeable (0-0.5), slightly noticeable (0.5-1.5), noticeable (1.5-3.0), and well visible (3.0-6.0) to great (6.0-12.0) [29, 30].

The Δ*E* values obtained for the HHP-treated (400-600 MPa/15 min) juices reached an average value of 2.59, indicating noticeable total colour changes after processing (Table 2). However, almost threefold higher Δ*E* values were noted in the juices subjected to LPT and HPT treatment compared to those in the pressurized samples. Similarly, it was found that the tomato and carrot juices subjected to heat processing (80°C/1 min) showed a greater variation in total colour compared to the HHP-treated juices (250 MPa/35°C/15 min) [6]. The changes in Δ*E* values observed, in the above cited report, for HHP-treated tomato and carrot juices did not exceed 15. In turn, the thermally treated samples presented distinctly higher values for this parameter. According to Dede and coauthors [6], these discrepancies may be attributed to the different effects of HHP and thermal treatment on the release of carotenoids from the protein complexes and/or the breakdown of phytochemicals in the treated material.

The results presented in Table 2 indicated that the heat-pasteurized juices showed approximately 13% (LPT sample) and 25% (HPT sample) decreases in *a*^∗^ values. They also showed an increase (by 14% and 27% on average) in the *L*^∗^ and *b*^∗^ parameters, respectively. In the study of Kelebek et al. [30], a similar drop (17%) in the *a*^∗^ value was noted in the hot break tomato paste. These authors explained that phenomenon as an increase in the redness of the tomato paste due to the evolution of the brown colour along with the processing time.

**Figure 1:**
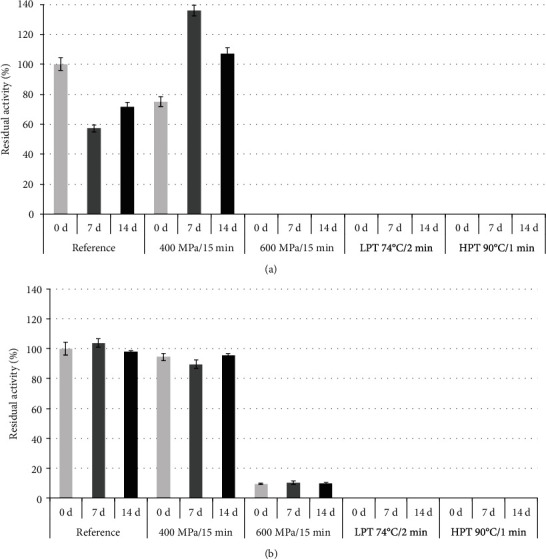
Activity of PPO (a) and POD (b) as analysed in the untreated, HHP-treated, and pasteurized (LPT and HPT) tomato juice after 0, 7, and 14 days of storage. The (%) residual activities ((*A*/*A*_0_) · 100) are presented, where *A*_0_ represents the initial enzyme activity determined in freshly prepared juice (*Δ*OD/min/g fw). Vertical bars indicate standard deviations of the means (*n* = 3). ^∗^ND: not detected.

**Figure 2:**
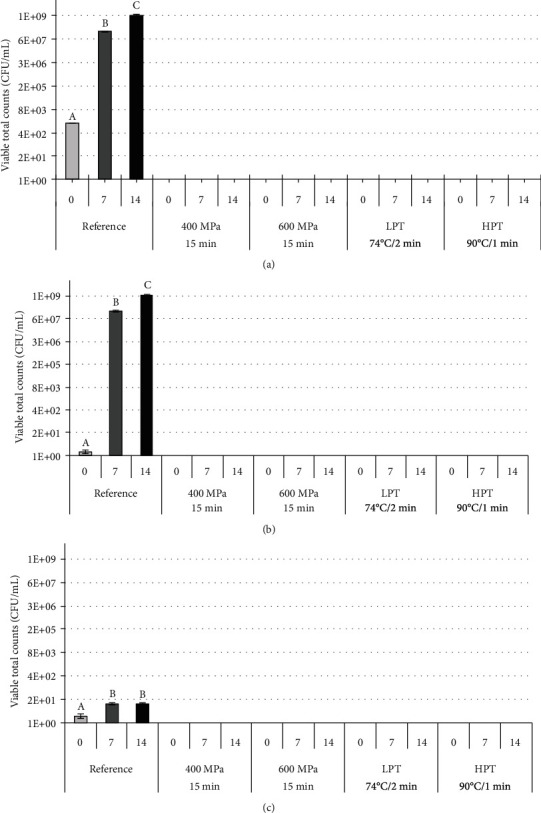
Average total bacterial count (a), total yeast count (b), and total mould count (c) as analysed in the untreated, HHP-treated, and pasteurized (LPT and HPT) tomato juice after storage for 0, 7, and 14 days. The mean values denoted with the same letter are not significantly different, *p* < 0.05. ^∗^Below the detection limit of <1 CFU/mL.

As expected, the untreated juice stored for 7 and 14 days demonstrated the most distinct changes in total colour. We may speculate that the enzyme (PPO, POD) activity found in the untreated juice (Figure 1) along with the microbial instability of the untreated juice (Figure 2) was responsible for the major observed colour changes, especially in the changes in the *a*^∗^ value. An interesting phenomenon was observed in the stored HHP-treated samples. The tested juices at 7 and 14 days of storage had 12% and 17% (on average) lower *b*^∗^ values, respectively, than the freshly pressurized juices. However, only a slight variation was noted in the *a*^∗^ values of the analysed samples. This suggests that the yellow colour was unstable in the stored HHP-treated juices. Compared to the HHP-treated juices, juices exposed to heat pasteurization demonstrated less advanced but still noticeable and visible changes in Δ*E* values after 7 and 14 days of storage, respectively. Following Marszałek and coauthors [31], we speculate that the processes combined with the storage time may disrupt the equilibrium between labile phytochemicals, leading to a transformation in their structure that, in turn, creates less or more advanced changes in the analysed colour parameters.

### 3.4. Activity of PPO and POD

The initial activity of PPO determined in the freshly prepared (untreated) tomato juices was lower (*A*_0_ = 0.14*Δ*OD/min/g fw) than the activity of POD (*A*_0_ = 35.70*Δ*OD/min/g fw). The initial activity of POD measured by Hernandez and Cano [32] in fresh tomato puree reached a similar value (40.41 *Δ*OD/min/g fw) as the data presented in our study. However, these authors obtained much higher PPO activity (0.79 *Δ*OD/min/g fw) than the value noted for our sample.

The effects of processing on the PPO and POD residual activity in tomato juices are shown in Figures 1(a) and 1(b). While the fresh juice treated with 400 MPa/15 min showed reduced (by 25%) PPO activity (Figure 1(a)), the POD activity did not change significantly after the processing applied (Figure 1(b)). The PPO was completely inactivated when the juice was exposed to 600 MPa for 15 min or subjected to thermal pasteurization (LPT/HPT). The complete inactivation of POD was also achieved after LPT and HPT treatment, whereas the treatment at 600 MPa/15 min reduced its activity by 90%. In contrast to our results, Marszałek and coauthors [13] found that PPO was more resistant to pressure (200-900 MPa) than POD. However, these authors studied the effect of processing on the initial activities of pure and isolated enzymes. Since the mechanism of enzyme inactivation under HPP was not fully explained, we could speculate that the differences in the chemical structure of PPO and POD are not the main determinants of their barostability and that the behaviour of the food matrix components and interaction between them should also be taken into account when considering the enzyme stability in the HHP-treated food system.

At 7 and 14 days of storage, a significant increase (by approximately 36% and 7%, respectively) in PPO activity was found for the juice treated with 400/MPa/15 min. An almost 200% increase in the residual activity of PPO was also observed by Sulaiman and Silva [33] for strawberry puree stored under frozen conditions for 30 days. However, these authors did not explain this finding. On the other hand, this phenomenon may be related to the fact that the structure of the cellular membranes was disturbed as a result of HHP processing of tomato material [32], which subsequently could have led to a gradual PPO release during sample storage. The POD activity determined in the freshly prepared juice as well as in the HHP-treated (400 MPa/15 min) juice remained at a similar level throughout the entire storage period (Figure 1(b)).

### 3.5. Microbiological Evaluation

The results of the total aerobic microbial count (TAMC), total yeast count (TYC), and total mould count (TMC) obtained for the analysed juices are presented in Figures 2(a)–2(c).

For the fresh reference juice, 10^3^ total bacteria were initially noted (Figure 2(a)). On days 7 and 14 of storage, the untreated juice contained 10^8^ and 10^9^ microorganisms/mL, respectively. A rapid increase in the yeast population (by nine orders of magnitude) was noted in untreated tomato juice at 14 days of storage (Figure 2(b)). In contrast, the growth of mould in the juice during the analysed storage period was reduced to 10^1^ microorganisms/mL (Figure 2(c)). This phenomenon could be related to the fact that yeast and mould have special nutritional requirements; thus, a competition effect might occur between them during the tested storage period. Moreover, the toxicity of yeast towards mould, due to the specific metabolites excreted by yeast, might also affect the mould population in the analysed juices [34].

The results clearly showed that the HHP (400–600 MPa/15 min), LPT, and HPT processes were equally effective in inhibiting the growth of microorganisms to a level below the limit of detection (<1 CFU/mL) during storage (Figures 2(a)–2(c)). Our results are in accordance with data presented by Plaza and coauthors [35], who also found a significant decrease (by 4 logarithmic units) in the total microbial count after tomato puree was exposed to 400 MPa for 15 min. As indicated by these authors, the processing parameters were sufficient to completely inactivate the yeast and mould populations. Similarly, the pressurization of tomato juice at 600 MPa for 1 min or its thermal processing at 95°C for 20 min also effectively reduced the total viable count below the detection limit < 1 CFU/mL [9].

### 3.6. Principal Component Analysis (PCA)

PCA was carried out based on all analysed samples and variables (total phenolic content (TPC), total phenolic index (TPI), antioxidant capacity (ABTS, FRAP), colour parameters (*L*, *a*, *b*, and Δ*E*), and enzyme activity (PPO/POD)) to characterize the structure and regularity in the relationships between variables and outcomes.

**Figure 3:**
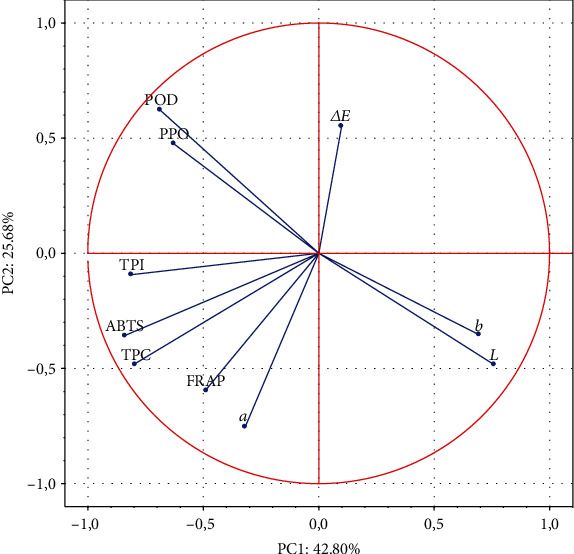
Principal component plot, variations in the parameters (TPC, TPI, ABTS, FRAP, *L*^∗^, *a*^∗^, *b*^∗^, Δ*E*, PPO, and POD) of untreated, HHP-treated, and pasteurized (HPT and LPT) tomato juice upon 0, 7, and 14 days of storage.

The first factor (PC1, 42.80%) was marked by high loadings on ABTS (-0.8385), TPI (-0.8122), and TPC (-0.7964), and the second factor (PC2, 25.68%) by high loadings on *a*^∗^ (-0.7540) and POD (0.6235). The factors with opposite signs exerted opposite effects (Figure 3). In the analysis, TPC, ABTS, and TPI were located close to one another due to the presence of significant and positive correlations. On the other hand, these parameters appeared to be weakly correlated with *a*^∗^. The location of the *a*^∗^ vector confirms its significant and negative impact only on PC2. The impact of *a*^∗^ on PC1 is rather minor (-0.3193). Similarly, the TPI vector has high negative loadings on PC1, but it has a negligible impact on PC2 (-0.0929). Thus, TPI is significantly correlated only with ABTS and TPC parameters. The correlation coefficients (Table 3) confirmed the relationships among the tested parameters. Our results are in accordance with other literature data [15, 23]. In the cited references, phenolic concentrations were positively and significantly correlated with the scavenging capacity of tomato fruit.

**Figure 4:**
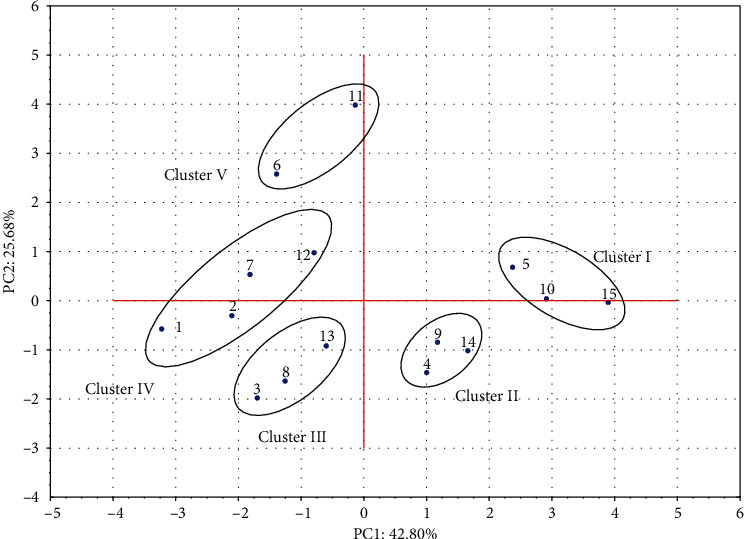
Score plot of PC1 vs. PC2 of the untreated, HHP-treated, and pasteurized (LPT and HPT) tomato juices during 0, 7, and 14 days of storage. Samples were coded as follows: untreated: 1, 6, and 11; 400 MPa/15 min: 2, 7, and 12; 600 MPa/15 min: 3, 8, and 13; LPT: 4, 9, and 14; and HPT: 5, 10, and 15. Numbers 1, 2, 3, 4, and 5 were assigned to the fresh samples, and the stored samples were coded as 6, 7, 8, 9, and 10 (7 days of storage) and 11, 12, 13, 14, and 15 (14 days of storage).

All the analysed samples were additionally described in the coordinate system of the first two principal components. The score plot generated by PCA classified and detected five different groups in the data structure (Figure 4). The nature of the differences between the obtained clusters resulted more from the processing than from the juice storage conditions. Regardless of the storage period, the samples treated with HPT formed cluster I. These samples had low TPC, TPI, ABTS, and FRAP values but high values of *L*^∗^ and *b*^∗^. A similar trend was observed in the LPT-treated and stored samples, which formed cluster II. Samples treated with 600 MPa for 15 min and stored for 0, 7, and 14 days were in cluster III and had high TPC, TPI, ABTS, FRAP, and *a*^∗^ values. Cluster IV included the juices treated with 400 MPa/15 min (fresh and stored) and the fresh, untreated sample. The composition of that cluster indicates the negligible impact of processing at 400 MPa/15 min on the quality parameters of the juice. As shown in Figure 4, the untreated juice stored for 7 and 14 days formed cluster V and had high PPO, POD, and Δ*E* values.

## 4. Conclusions

Compared to the heat-pasteurized juices stored for 14 days, the juice exposed to 600 MPa for 15 min showed superior quality. Samples treated with 600 MPa for 15 min and stored for 0, 7, and 14 days had high TPC, TPI, ABTS, FRAP, and *a*^∗^ values. As demonstrated, these tested samples at the end of the storage period retained 90% and 95% of their polyphenol content and antioxidant capacity, respectively. As in the case of pasteurization, juice processing at 600 MPa for 15 min clearly reduced the activity of food-spoiling enzymes (PPO, POD) as well as the microbial count. The obtained results showed that TPC was significantly and positively correlated with TPI, ABTS, and FRAP parameters. The pressurized and stored juices demonstrated high stability in their *a*^∗^ values, while variations in yellow colour were observed.

As indicated by the results presented, HHP (600 MPa/15 min) without the use of temperature allowed to preserved polyphenol content and antioxidant capacity of the tomato juice. The preservation of a high nutritional value in the HHP-treated juice with a simultaneous complete inactivation of undesirable microflora and enzymes allows to demonstrate the superiority of the HHP process over the heat treatment.

## Figures and Tables

**Table 1:** Effect of processing on the total phenolic content (TPC), total phenolic index (TPI), and antioxidant capacity (ABTS, FRAP) in untreated, HHP-treated, and pasteurized (LPT and HPT) tomato juice after 0, 7, and 14 days of storage.

Sample		Storage time (days)	
	0	7	14
TPC (mg GAE/g dm)			
Untreated/reference	7.08 ± 0.16^Bc∗^	6.06 ± 0.35^Bb^	5.27 ± 0.35^Ba^
400 MPa/15 min	6.94 ± 0.20^Bc^	6.13 ± 0.39^Bb^	5.48 ± 0.42^BCa^
600 MPa/15 min	7.42 ± 0.35^Bb^	7.78 ± 0.56^Cb^	6.65 ± 0.34^Da^
LPT (74°C/2 min)	5.61 ± 0.71^Aa^	5.94 ± 0.74^ABa^	5.88 ± 0.28^Ca^
HPT (90°C/1 min)	4.91 ± 0.10^Aa^	5.05 ± 0.61^Aa^	4.47 ± 0.27^Aa^
TPI (*μ*g/100 g dm)			
Untreated/reference	153.57 ± 2.29^Bb^	143.67 ± 2.98^Bab^	124.67 ± 1.61^Ca^
400 MPa/15 min	150.45 ± 1.90^Bb^	143.80 ± 2.05^Bab^	136.99 ± 1.88^Da^
600 MPa/15 min	164.37 ± 2.04^Bb^	140.57 ± 2.95^Bab^	134.46 ± 1.90^Da^
LPT (74°C/2 min)	144.24 ± 1.95^ABb^	144.00 ± 1.92^Bb^	77.67 ± 1.09^Ba^
HPT (90°C/1 min)	125.87 ± 1.72^Ac^	85.85 ± 0.79^Ab^	61.55 ± 0.93^Aa^
ABTS (*μ*mol TE/g dm)			
Untreated/reference	8.44 ± 0.27^Bc^	7.81 ± 0.10^Bb^	6.94 ± 0.13^Ba^
400 MPa/15 min	8.43 ± 0.39^Bb^	7.47 ± 0.38^Ba^	7.29 ± 0.40^BCa^
600 MPa/15 min	8.33 ± 0.56^Bb^	8.51 ± 0.26^Cb^	7.66 ± 0.33^Ca^
LPT (74°C/2 min)	7.71 ± 0.79^Ba^	7.41 ± 0.18^Ba^	7.62 ± 0.28^Ca^
HPT (90°C/1 min)	6.24 ± 0.36^Ab^	6.64 ± 0.22^Ab^	5.34 ± 0.24^Aa^
FRAP (*μ*mol TE/g dm)			
Untreated/reference	38.79 ± 0.64^Dc^	35.15 ± 0.71^Bb^	30.52 ± 0.93^Aa^
400 MPa/15 min	34.52 ± 0.83^Bb^	35.62 ± 0.76^Bb^	32.84 ± 0.73^Ba^
600 MPa/15 min	37.90 ± 0.71^Cb^	36.66 ± 0.78^Ca^	36.11 ± 0.86^Ca^
LPT (74°C/2 min)	39.30 ± 0.39^Dc^	32.22 ± 0.73^Aa^	34.14 ± 0.79^Bb^
HPT (90°C/1 min)	33.36 ± 0.54^Ab^	31.21 ± 0.76^Aa^	33.93 ± 0.96^Bb^

The results are expressed as the mean ± SD, *n* = 6. ^∗^Uppercase letters indicate differences between treatment types (in columns) on particular days of storage, whereas lowercase letters indicate differences between storage times for individual samples (in lines) (*p* ≤ 0.05).

**Table 2:** Effect of processing on colour parameters measured in untreated, HHP-treated, and pasteurized (LPT and HPT) tomato juice after 0, 7, and 14 days of storage.

Sample		Storage time (days)	
	0	7	14
*L* ^∗^			
Untreated/reference	29.39 ± 0.13^Aa∗∗^	29.85 ± 0.01^Ab^	29.22 ± 0.01^Aa^
400 MPa/15 min	30.65 ± 0.13^Ba^	30.90 ± 0.01^Bb^	32.30 ± 0.05^Bc^
600 MPa/15 min	30.30 ± 0.10^Ba^	33.43 ± 0.03^Cc^	32.94 ± 0.06^Cb^
LPT (74°C/2 min)	34.80 ± 0.13^Cc^	33.98 ± 0.03^Eb^	33.24 ± 0.03^Da^
HPT (90°C/1 min)	33.45 ± 0.10^Ca^	33.67 ± 0.02^Db^	34.56 ± 0.03^Dc^
*a* ^∗^			
Untreated/reference	23.15 ± 0.18^Cc^	12.71 ± 0.04^Ab^	12.18 ± 0.04^Aa^
400 MPa/15 min	24.04 ± 0.19^Ca^	23.28 ± 0.01^Ea^	23.44 ± 0.05^Ca^
600 MPa/15 min	23.61 ± 0.21^Cb^	22.30 ± 0.05^Da^	23.98 ± 0.02^Cc^
LPT (74°C/2 min)	20.08 ± 0.20^Ba^	19.94 ± 0.04^Ca^	20.94 ± 0.04^Bb^
HPT (90°C/1 min)	17.20 ± 0.21^Aa^	17.81 ± 0.04^Bb^	20.82 ± 0.05^Bc^
*b* ^∗^			
Untreated/reference	24.34 ± 0.20^Ab^	22.23 ± 0.02^Aa^	22.24 ± 0.03^Ba^
400 MPa/15 min	26.48 ± 0.17^Bc^	24.38 ± 0.02^Bb^	23.67 ± 0.02^Ca^
600 MPa/15 min	26.66 ± 0.16^Bc^	22.19 ± 0.03^Ab^	20.37 ± 0.03^Aa^
LPT (74°C/2 min)	30.31 ± 0.20^Db^	28.71 ± 0.01^Ca^	28.93 ± 0.04^Da^
HPT (90°C/1 min)	28.76 ± 0.18^Ca^	30.15 ± 0.03^Db^	30.32 ± 0.05^Eb^
Δ*E*			
Untreated/reference	0.00 ± 0.00^Aa^	10.66 ± 0.09^Db^	11.17 ± 0.06^Db^
400 MPa/15 min	2.69 ± 0.20^Bab^	2.32 ± 0.03^Ba^	3.36 ± 0.05^Bb^
600 MPa/15 min	2.54 ± 0.19^Ba^	5.88 ± 0.04^Cb^	6.83 ± 0.04^Cc^
LPT (74°C/2 min)	8.62 ± 0.20^Cb^	1.86 ± 0.02^ABa^	2.15 ± 0.04^Aa^
HPT (90°C/1 min)	8.45 ± 0.19^Cc^	1.54 ± 0.05^Aa^	4.09 ± 0.08^Bb^

The results are expressed as the mean ± SD, *n* = 3. ^∗^Three independent measurements: *L*^∗^ (lightness), *a*^∗^ (redness), and *b*∗ (yellowness). Δ*E* is the colour difference between the control and processed tomato juices. ^∗∗^Uppercase letters indicate differences between treatment types (in columns) on particular days of storage, whereas lowercase letters indicate differences between storage times for individual samples (in lines) (*p* ≤ 0.05).

**Table 3:** Pearson correlation coefficient indicating the relationship between the tested parameters in untreated, HHP-treated, and pasteurized (LPT and HPT) tomato juice after 0, 7, and 14 days of storage.

Correlation	TPC	TPI	ABTS	FRAP
TPC	—	0.668^∗∗^	0.914^∗∗^	0.609^∗^
TPI	0.668^∗∗^	—	0.735^∗∗^	0.484
ABTS	0.914^∗∗^	0.735^∗∗^	—	0.581^∗^
FRAP	0.609^∗^	0.484	0.581^∗^	—
Δ*E*	-0.247	0.080	-0.178	-0.090
*L*	-0.394	-0.500	-0.450	-0.077
*a*	0.505	0.213	0.375	0.491
*b*	-0.495	-0.461	-0.439	-0.076
PPO	0.101	0.368	0.228	-0.009
POD	0.163	0.417	0.310	-0.042

^∗^Correlation is significant at the 0.05 level. ^∗∗^Correlation is significant at the 0.01 level.

## Data Availability

Data supporting the results were preserved at the Institute of Animal Reproduction and Food Research, Polish Academy of Sciences, in Olsztyn, Poland, and they are available from the corresponding author upon request.
